# A non-surgical approach to the management of exposure keratitis due to facial palsy by using mini-scleral lenses: Erratum

**DOI:** 10.1097/MD.0000000000007118

**Published:** 2017-06-02

In the article, “A non-surgical approach to the management of exposure keratitis due to facial palsy by using mini-scleral lenses”,^[1]^ which appeared in Volume 96, Issue 6 of *Medicine*, Victor Zaki's email should appear as victorzaki@gmail.com.
